# Advisory Committee on Immunization Practices Recommended Immunization Schedule for Adults Aged 19 Years or Older — United States, 2024

**DOI:** 10.15585/mmwr.mm7301a3

**Published:** 2024-01-11

**Authors:** Neil Murthy, A. Patricia Wodi, Veronica V. McNally, Matthew F. Daley, Sybil Cineas

**Affiliations:** ^1^Immunization Services Division, National Center for Immunization and Respiratory Diseases, CDC; ^2^Franny Strong Foundation, West Bloomfield, Michigan; ^3^Institute for Health Research, Kaiser Permanente Colorado, Aurora, Colorado; ^4^The Warren Alpert Medical School of Brown University, Providence, Rhode Island.

At its October 2023 meeting, the Advisory Committee on Immunization Practices* (ACIP) approved the Recommended Adult Immunization Schedule for Ages 19 Years or Older, United States, 2024. The adult immunization schedule, which can be found on the CDC immunization schedule website (https://www.cdc.gov/vaccines/schedules), is published annually to consolidate and summarize updates to ACIP recommendations on the vaccination of adults and to assist health care providers in implementing current ACIP recommendations. The 2024 immunization schedule includes several changes to the cover page, tables, notes, and appendix from the 2023 immunization schedule.^†^ In addition, the 2024 adult immunization schedule includes a new addendum section that summarizes new or updated ACIP recommendations that will occur before the next annual update to the adult immunization schedule. Health care providers are advised to use the cover page, tables, notes, appendix, and addendum together to determine recommended vaccinations for patient populations.

This adult immunization schedule is recommended by ACIP (https://www.cdc.gov/vaccines/acip) and approved by CDC (https://www.cdc.gov), the American College of Physicians (https://www.acponline.org), the American Academy of Family Physicians (https://www.aafp.org), the American College of Obstetricians and Gynecologists (https://www.acog.org), the American College of Nurse-Midwives (https://www.midwife.org), the American Academy of Physician Associates (https://www.aapa.org), the American Pharmacists Association (https://www.pharmacist.com), and the Society for Healthcare Epidemiology of America (https://shea-online.org). 

ACIP’s recommendations on the use of each vaccine are developed after in-depth reviews of vaccine-related data, including disease epidemiology and societal impacts, vaccine efficacy and effectiveness, vaccine safety, quality of evidence, feasibility of program implementation, impact on health equity, and economic analyses of immunization policy (*1*,*2*). Health care providers should be aware that changes in recommendations for specific vaccines occur between these annual updates to the adult immunization schedule.^§^ Such changes will be summarized in the new addendum section; however, health care providers are encouraged to refer to ACIP recommendations for detailed guidance on the use of each vaccine (https://www.cdc.gov/vaccines/hcp/acip-recs). An online version of the 2024 adult immunization schedule and instructions for downloading the schedule app to use on mobile devices are available on the immunization schedule website (https://www.cdc.gov/vaccines/schedules). The use of vaccine trade names in this report and in the adult immunization schedule is for identification purposes only and does not imply endorsement by ACIP or CDC.

## Changes in the 2024 Adult Immunization Schedule

Vaccine-specific changes in the 2024 immunization schedule for adults aged ≥19 years include new and updated recommendations for respiratory syncytial virus vaccines (RSV) (*3*), influenza vaccines (*4*), COVID-19 vaccines (*5*), inactivated poliovirus vaccine (IPV) (*6*), Mpox vaccine (Mpox) (https://www.cdc.gov/vaccines/acip/meetings/downloads/slides-2023-10-25-26/04-MPOX-Rao-508.pdf), and meningococcal serogroups A, B, C, W, Y pentavalent vaccine (MenACWY-TT/MenB-FHbp) (https://www.cdc.gov/vaccines/acip/recommendations.html). Any reference to meningococcal serogroups A, C, W, Y polysaccharide diphtheria toxoid conjugate vaccine (MenACWY-D [Menactra]) was removed from the schedule because this product is no longer distributed in the United States. Other changes include clarification of the recommendations for hepatitis A vaccine (HepA), hepatitis B vaccine (HepB), human papillomavirus vaccine (HPV), measles, mumps, and rubella vaccine (MMR), pneumococcal vaccines, and tetanus, diphtheria, and pertussis vaccine (Tdap).

### Cover page

• A fifth step in the “How to Use the Adult Immunization Schedule” box was added directing health care providers to review the new addendum section that lists new or updated ACIP recommendations that occur before the next annual update to the adult immunization schedule.

• Information on injury claims, travel vaccine recommendations and a hyperlink to the 2024 child and adolescent immunization schedule was removed from the Cover Page and moved to a new “Additional Information” section on the first page of the Notes. This was done to harmonize presentation of this information with the 2024 child and adolescent immunization schedule.

• Mpox (Jynneos), pentavalent meningococcal vaccine (MenACWY-TT/MenB-FHbp [Penbraya]), and RSV vaccines (Abrysvo [Pfizer Inc.] and Arexvy [GSK]) were added to the table of vaccine abbreviations and trade names.

• MenACWY-D (Menactra) was removed from the table of vaccine abbreviations and trade names because it is no longer distributed in the United States, and any remaining doses of this product expired in October 2023.

• The bivalent mRNA COVID-19 vaccines were removed from the table of vaccine abbreviations and trade names because current mRNA COVID-19 vaccines are all monovalent, and the bivalent mRNA COVID-19 vaccines used in the United States during 2022–2023 are no longer recommended.

### Table 1 (Routine Immunization Schedule)

• **COVID-19 row:** The text overlay was revised to reflect updated vaccination recommendations. This text overlay now states, “1 or more doses of updated (2023–2024 Formula) vaccine.”

• **RSV row:** The RSV vaccination is a new addition to this table. The color of this row is purple for adults aged 19–49 years, with overlaying text “seasonal administration during pregnancy,” reflecting the recommendation for the use of Abrysvo (Pfizer Inc.) during 32–36 weeks’ gestation. The row is light blue for adults aged ≥60 years, indicating that the recommendation for RSV vaccination with either Abrysvo (Pfizer Inc.) or Arexvy (GSK) among adults aged ≥60 years is based on shared clinical decision-making.

• **Mpox row**: A new row was added for Jynneos, with a purple bar across all ages reflecting the risk-based recommendation for Mpox vaccination.

### Table 2 (Immunization by Medical Indication Schedule)

• A header sentence was added to Table 2 stating that medical conditions or indications are often not mutually exclusive and advising health care providers to review all relevant columns in the table if multiple conditions or indications are present.

• **Legend:** The definitions of the yellow, purple, and gray colors in the legend were revised. The new definitions of these colors are intended to be more focused and narrower, such that the recommendation for vaccination based on that medical indication is more readily apparent. In addition, brown was introduced as a new legend color, indicating that additional doses of vaccine might be necessary based on medical condition or other indication. To account for these revised color definitions, many of the vaccine rows in Table 2 were recolored.

• **HepB row:** Under the diabetes column, a blue bar was added to indicate that the recommendation for vaccination for persons aged ≥60 years with diabetes is based on shared clinical decision-making.

• **RSV row:** The RSV vaccination is a new addition to this table. For use during pregnancy, the color is yellow with overlaying text of “seasonal administration” to indicate that the use of Abrysvo (Pfizer Inc.) in pregnancy is based on RSV seasonality. For the rest of the medical indications listed, the color is light blue reflecting that the recommendation for vaccination among adults aged ≥60 years is based on shared clinical decision-making.

• **Mpox row:** A new row was added for Jynneos. Across all medical indications listed, the entire row is purple reflecting the risk-based recommendation for Mpox vaccination. In the pregnancy column, an overlaying text “See Notes” was added to encourage health care providers to review the pregnancy bullet in the Mpox vaccination notes.

### Vaccine Notes

The notes for each vaccine are presented in alphabetical order. Edits have been made throughout the Notes section to harmonize language, to the greatest extent possible, with that in the child and adolescent schedule.

• A new “Additional Information” section now begins the Notes section of the 2024 adult immunization schedule. This section mirrors the “Additional Information” section in the Notes section of the 2024 child and adolescent immunization schedule and contains similar information. Bullets that were previously on the Cover Page (such as injury claims and travel vaccine recommendations, etc.) have now been incorporated into the new “Additional Information” section of the Notes section. The text for vaccine injury compensation was revised to add Mpox and RSV to the list of vaccines not covered by the National Vaccine Injury Compensation Program. Mpox is covered by the Countermeasures Injury Compensation Program.

• **COVID-19**: All adults are now recommended to receive at least 1 dose of an updated (2023–2024 Formula) COVID-19 vaccine. The number of doses needed and intervals between doses might vary based on a patient’s previous vaccination history, immunocompromise status, and the vaccine product used. In addition, the COVID-19 notes section is divided into a “Routine vaccination” section that describes the vaccination recommendations for the general population and a “Special situations” section that describes the vaccine recommendations for persons who are moderately or severely immunocompromised.

• **HepA**: To better align the language with ACIP policy, the bullet in the “Routine vaccination” section was revised to, “Any person who is not fully vaccinated and requests vaccination.” The HepA vaccine regimen is described in detail later in that bullet.

• **HepB**: In the “Routine vaccination” section, additional context and details were added to the bullets describing the risk-based vaccination recommendation for persons aged ≥60 years. In addition, a note was added at the end of the “Routine vaccination” section describing the shared clinical decision-making recommendation for persons aged ≥60 years with diabetes.

• **HPV:** In the “Routine vaccination” section, the guidance on interrupted schedules was removed because that information is presented on the Cover Page. Age ranges were reordered to be in chronological order. In addition, to improve clarity, the words “of any valency” were added to the bullet, “No additional dose recommended when any HPV vaccine series *of any valency* has been completed using the recommended dosing intervals.” Lastly, a link to a resource was added to assist health care providers with shared clinical decision-making recommendations for HPV vaccination.

• **Influenza**: A hyperlink to the 2023–24 influenza recommendations and a bullet regarding the 2024–25 influenza recommendations were added. In the “Special situations” section, all bullets that discuss history of egg allergy were removed, and a note was added at the end of the “Special situations” section stating that persons with a history of egg allergy can be vaccinated with any influenza vaccine indicated for the recipient’s age and health status (*4*). Finally, the bullet describing Guillain-Barré syndrome was removed because this information is presented in the Appendix section on contradictions and precautions.

• **MMR**: Minor changes were made to the “Routine vaccination” section to improve language clarity.

• **Meningococcal**: All references to Menactra were removed because this product is no longer distributed in the United States. A link to a resource was added to assist health care providers with shared clinical decision-making recommendations for MenB vaccination. Lastly, information about the use of the newly licensed pentavalent meningococcal vaccine (Penbraya) is provided at the end of the meningococcal notes section.

• **Mpox:** Mpox vaccination is a new addition to the Notes section of the adult immunization schedule. Risk factors that warrant routine Jynneos vaccination are listed. Bullets about the use of Jynneos among health care providers and in pregnant persons are provided at the end of the Mpox notes section.

• **Pneumococcal:** Minor edits were made throughout the “Routine vaccination” and “Special situations” sections to provide clarity on the guidance and minimum intervals between doses of pneumococcal vaccines.

• **Poliovirus**: Additional context was added to the “Routine vaccination” section. This section now calls for adults who are known or suspected to be unvaccinated or incompletely vaccinated to complete the 3-dose IPV primary vaccination series. A statement was added stating that most adults who were born and raised in the United States can assume that they were vaccinated against polio as children. The “Special situations” section describes administering a one-time, lifetime IPV booster dose to adults who have completed the primary series and who are at increased risk for exposure to poliovirus.

• **RSV**: A new RSV notes section was added this year. The section begins with a “Routine vaccination” section that describes the use of Abrysvo (Pfizer Inc.) in pregnant persons during 32–36 weeks’ gestation from September through January in most of the continental United States. In addition, a sub-bullet was added stating that either maternal RSV vaccination or infant immunization with nirsevimab (RSV monoclonal antibody) is recommended to prevent respiratory syncytial virus lower respiratory tract infection in infants. A note was added at the end of the RSV notes section to acknowledge that certain jurisdictions might have RSV seasonality that differs from most of the continental United States, and that providers should follow guidance from public health authorities regarding the timing of maternal RSV vaccine administration, based on local RSV seasonality. The “Special situations” section describes the shared clinical decision-making recommendation for vaccination of persons aged ≥60 years; either Abrysvo (Pfizer Inc) or Arexvy (GSK) may be used. In addition, a link to a resource was added to assist health care providers with shared clinical decision-making recommendations for RSV vaccination. Finally, a note was added that lists risk factors and medical conditions that health care providers should consider when thinking through a patient’s risk for severe RSV disease and potential benefit from vaccination.

• **Tdap**: A note was added at the end of the Tdap section to clarify that a dose of Tdap received at age 10 years may be counted as the adolescent dose routinely recommended at age 11–12 years.

### Appendix (Contraindications and Precautions)

• The header sentence of the Appendix was revised to include all the sources used to create the Appendix.

• **COVID-19 row:** Two new rows for COVID-19 vaccines were added describing the contraindications and precautions to COVID-19 vaccination. The first row lists the contraindications and precautions to mRNA vaccines (Pfizer-BioNTech and Moderna), and the second row lists the contraindications and precautions to the protein subunit vaccine (Novavax).

• **Hib row**: In the “Contraindicated or Not Recommended” column, the bullet describing history of severe allergic reaction to dry natural latex was removed because vials of Hib products no longer contain latex.

• **Meningococcal rows**: All references to Menactra were removed because this product is no longer distributed in the United States. Contraindications and precautions to vaccination with the new pentavalent meningococcal vaccine (MenACWY-TT/MenB-FHbp [Penbraya]) were added.

• **Mpox row**: A new row for Mpox was added describing the contraindications and precautions to Mpox vaccination.

• **RSV row**: A new row for RSV was added describing the contraindications and precautions to RSV vaccination.

### Addendum

• A new addendum section was added to the adult immunization schedule to summarize new and updated ACIP recommendations that occur before the next annual update to the adult immunization schedule.

## Additional Information

The Recommended Adult Immunization Schedule, United States, 2024, is available at https://www.cdc.gov/vaccines/schedules/hcp/adult.html, and in the *Annals of Internal Medicine* (*7*). The full ACIP recommendations for each vaccine are also available at https://www.cdc.gov/vaccines/hcp/acip-recs/index.html. All vaccines identified in Tables 1 and 2 (except Zoster vaccine) also appear in the Recommended Immunization Schedule for Children and Adolescents, United States, 2024 (https://www.cdc.gov/vaccines/schedules/hcp/imz/child-adolescent.html). For vaccines that appear in both the adult immunization schedule and the child and adolescent immunization schedule, the language in both schedules has been harmonized to the greatest extent possible.

## Footnotes

* Recommendations for routine use of vaccines in adults are developed by ACIP, a federal advisory committee chartered to provide expert external advice and guidance to the CDC director on use of vaccines and related agents for the control of vaccine-preventable diseases in the civilian population of the United States. Recommendations for routine use of vaccines in adults are harmonized to the greatest extent possible with recommendations made by the American Academy of Pediatrics, the American Academy of Family Physicians, and the American College of Obstetricians and Gynecologists. ACIP recommendations become official agency guidelines once the recommendation has been adopted by the CDC Director. Additional information about ACIP is available at https://www.cdc.gov/vaccines/acip/.

† Past immunization schedules are available at https://www.cdc.gov/vaccines/schedules/hcp/schedule-related-resources.html.

§ CDC encourages organizations to use syndication as a more reliable method for displaying the most current and accurate immunization schedules on an organization's website rather than copying these schedules to their websites. Use of content syndication requires a one-time step that ensures an organization’s website displays current schedules as soon as they are published or revised; instructions for the syndication code are available on CDC’s website (https://www.cdc.gov/vaccines/schedules/resource-library/syndicate.html). CDC also offers technical assistance for implementing this form of content syndication (requests can be e-mailed to ncirdwebteam@cdc.gov).
